# Role of T1 Pelvic Angle in Assessing Sagittal Balance in Outpatients With Unspecific Low Back Pain

**DOI:** 10.1097/MD.0000000000002964

**Published:** 2016-03-07

**Authors:** Mingyuan Yang, Changwei Yang, Zhengfang Xu, Ziqiang Chen, Xianzhao Wei, Jian Zhao, Jie Shao, Guoyou Zhang, Yingchuan Zhao, Haijian Ni, Yushu Bai, Xiaodong Zhu, Ming Li

**Affiliations:** From the Department of Orthopedics (MY, CY, ZC, XW, JZ, JS, GZ, YZ, HN, YB, XZ, ML), Changhai Hospital, Second Military Medical University, Shanghai; and Department of Obstetrics and Gynecology (ZX), Affiliated Hospital of Jiangsu University, Jiangsu, China.

## Abstract

The aim of the study was to explore the significance of T1 pelvic angle (TPA) for assessment of sagittal balance in a cohort of Chinese patients with unspecific low back pain.

TPA has been commonly used to assess sagittal balance in adult spinal deformity. However, whether TPA could be used to assess sagittal balance in patients with unspecific low back pain effectively remains unanswered.

Medical records of outpatients with unspecific low back pain who received treatment in our outpatient clinic between September 2013 and November 2014 were reviewed. Demographic data and radiographic data were collected. Correlation coefficients between TPA and other sagittal parameters were analyzed, and the intraclass correlation coefficient (ICC) analysis was performed to assess the inter- and intra-observer reliability of TPA. Patients were divided into 2 groups according to whether they were well-aligned (TPA ≤ 20°) or poorly aligned (TPA > 20°), and then demographic and sagittal parameters were compared between the 2 groups of patients.

A total of 97 patients with unspecific low back pain were included in this study. The inter- and intraobserver reliability of the TPA measure had excellent agreement (ICC = 0.985 and 0.919, respectively). There were significant correlations between TPA and age, LL, PT, PI, T1SPI, SVA, and NRS (all *P* < 0.05). Of the 38 well-aligned patients in Group A, SVA was ≤5 cm in 33 (86.84%) patients and >5 cm in the other 5 (13.16%) patients, and of the 59 poorly aligned patients in Group B, SVA was >5 cm in 42 (71.19%) patients and ≤5 cm in the other 17 (28.81%) patients. There were significant differences in age, LL, SS, PT, PI, T1SPI, SVA, and NRS between the 2 groups of patients, but no significant difference was observed in TK and TL.

TPA could be used to assess sagittal balance in outpatients with unspecific low back pain effectively.

## INTRODUCTION

An increasing number of studies have suggested that low back pain is associated with sagittal balance,^1^ and therefore sagittal parameters have been the great concern during the preoperative assessment of sagittal deformity, surgical decision-making, and preparation of the surgical procedure, knowing that these parameters are significantly correlated with health-related quality of life measures.^2,3^ Traditionally, spinal measurements including the sagittal vertical axis (SVA), the C7 plumb line/sacro-femoral distance ratio (C7/SFD) and T1 spinopelvic inclination (T1SPI), and pelvic parameters including pelvic tilt (PT), pelvic incidence (PI), and sacral slope (SS) are used to assess the global sagittal balance and pelvic retroversion, respectively. However, none of these parameters can well represent the whole spinopelvic balance and the interactions between the spinal and pelvic parameters. Therefore, it is necessary to use sagittal parameters to understand the true extent of sagittal balance of a particular patient.^4,5^

**FIGURE 1:**
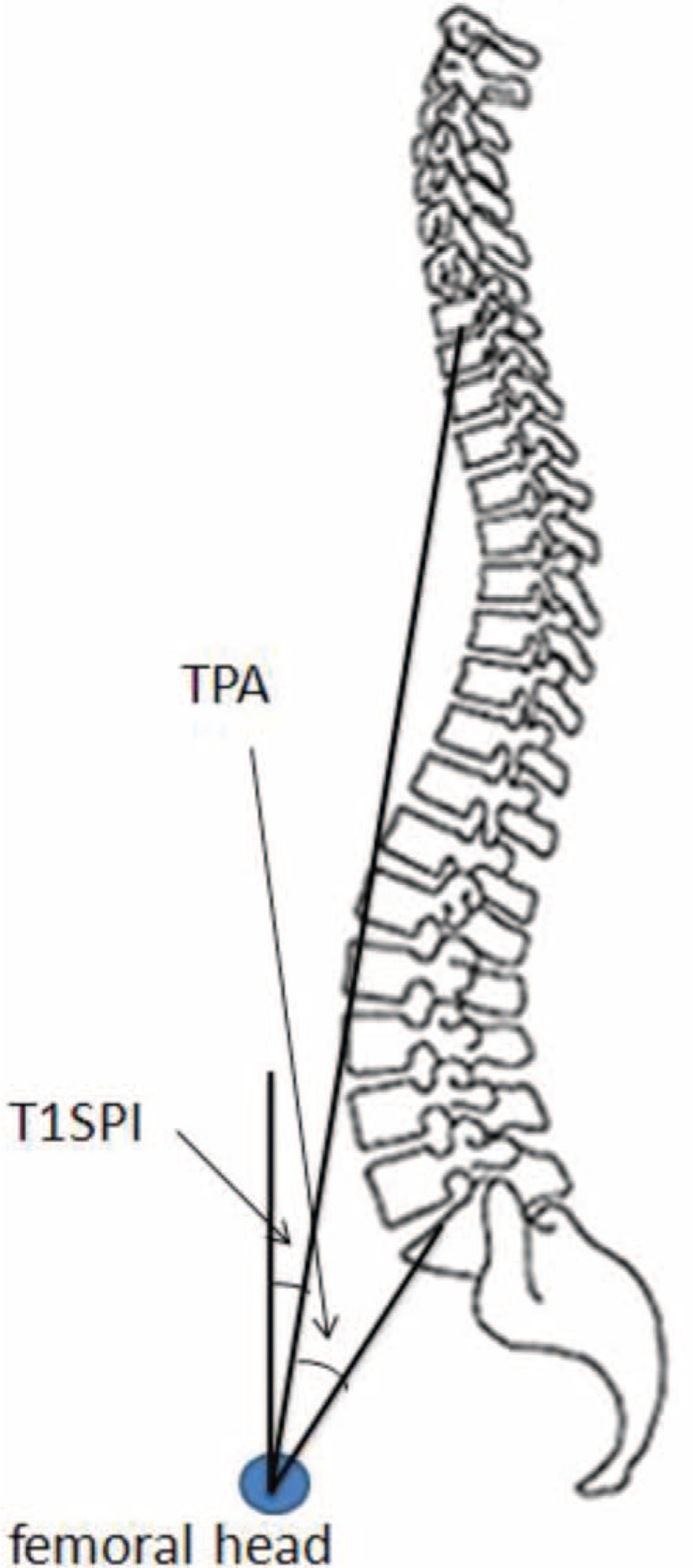
Schematic representation of the TPA and T1SPI. TPA is defined as the angle between the line from the femoral headaxis to the centroid of T1 and the line from the femoral head axis to the middle of the S1 endplate. T1SPI is defined as the angle between the vertical plumbline and the line drawn from the vertebral body centroid of T1 and the centroid of the bicoxofemoral axis. T1SPI = T1 spinopelvic inclination, TPA = T1 pelvic angle.

Recently, the T1 pelvic angle (TPA, the angle between the line from the femoral head axis to the centroid of T1 and the line from the femoral head axis to the middle of the S1 endplate), a novel radiographic measure of global sagittal deformity proposed by Protopsaltis et al,^6^ has been thought to be significantly related to both PT and SVA, and could well represent both spinal and pelvic parameters in the sagittal plane (Figure 1, the angle between the line from the femoral head axis to the centroid of T1 and the line from the femoral head axis to the middle of the S1 endplate). According to Protopsaltis et al, TPA measurement of sagittal deformity is independent of many postural compensatory mechanisms and therefore can be used as a preoperative planning tool, given a target TPA of <14°. However, only a small number of studies^5,6^ used this novel measurement in Caucasian patients with adult degenerative scoliosis (ADS). In addition, these studies mainly focused on whether TPA could effectively evaluate sagittal deformity and assess radiological and clinical outcomes longitudinally.^5,7^ To the best of our knowledge, no study has been conducted to determine whether TPA could be used to assess sagittal balance in Chinese patients with unspecific low back pain effectively, knowing that the spinal and pelvic parameters are significantly different between populations with different ethnicity backgrounds.^8^

The aim of this retrospective study was to investigate the feasibility of using TPA to measure sagittal balance in Chinese patients with unspecific low back pain and explore its correlation with health measures and other sagittal parameters in these patients.

## MATERIALS AND METHODS

### Patient Recruitment

Included in this retrospective study were patients with unspecific low back pain who sought medical help in our outpatient clinic between September 2013 and November 2014. Inclusion criteria were patients with unspecific (mechanical and nonradicular) low back pain for >3 months (Patients were identified using International Classification of Diseases 9th Revision, ICD-9).^9^ Standing full-spine lateral radiography was performed to obtain sufficient sagittal parameters from each patient. The exclusion criteria were (1) patients with lumbar fracture, lumbar spondylolisthesis, lumbar tumors, or intervertebral infection; (2) patients with neurological and neuromuscular diseases; (3) patients with previous inner ear infection or vestibular disorder with balance disturbance who were unable to stand independently without assistance; and (4) patients without sufficient radiographic parameters or with previous histories of spinal or pelvic surgery. Patients whose femoral head or T1 superior endplate could not be measured on the lateral radiographs were also excluded. This study was approved by the institutional review board of Changhai Hospital of the Second Military Medical University (Shanghai, China). Written informed consent was obtained from all patients involved in this study.

### Data Collection

Demographic and radiographic parameters were collected and measured by 2 individual surgeons, including patients’ age, TPA (the angle between the line from the femoral head axis to the centroid of T1 and the line from the femoral head axis to the middle of the S1 endplate), thoracic kyphosis,^10^ lumbar lordosis,^10^ thoracolumbar kyphosis (TL),^10^ sacral slope (SS),^11^ pelvic incidence (PI),^12^ pelvic tilt (PT),^11^ T1 spinopelvic inclination (T1SPI, the angle between the vertical plumbline and the line drawn from the vertebral body center of T1 and the center of the bicoxofemoral axis), and SVA (sagittal vertical axis).^10^ The parameters were measured twice at a 1-week interval for intraclass correlation coefficient (ICC) analysis. Numeric rating scales (NRS, 0–10) were used to assess the unspecific low back pain.^13^ ICC analysis was performed to assess the inter- and intraobserver reliability of TPA, and the correlation coefficient between TPA and other sagittal parameters was explored. The severe deformity threshold for TPA was 20° as proposed in Ryan et al,^5^ based on which the patients were divided into the well-aligned group (Group A, TPA ≤ 20°) and poorly aligned group (Group B, TPA > 20°). Then, demographic and sagittal parameters were compared between the 2 groups.

### Statistical Analysis

The data were analyzed using SPSS 17.0 statistics software (SPSS Inc, Chicago, IL). Descriptive statistics were listed in the form of mean and standard deviation (SD), which was used in the statistical analysis. Comparisons of each parameter between Group A and Group B were performed using independent 2-sampled *t* test. TPA and its correlations with other sagittal parameters were analyzed by the correlation coefficient test. ICC analysis was performed to assess the interobserver antraobserver reliability of TPA using a 2-way random effects model. The ICC value >0.75 was considered acceptable agreement/reproducibility/reliability for a research tool.^14^ Statistical significance was set at a level of *P* value of <0.05.^12^

## RESULTS

A total of 97 patients with a mean age of 67.37 years (range: 41–92 years) were included in this study. TPA, T1SPI, TK, LL, TL, SS, PT, PI, and SVA ranged from 1° to 51° with a median of 23.04°, −10° to 12° with a median of −2.59°, −13° to 64° with a median of 27.96°, −9° to 77° with a median of 36.34°, −62° to 23° with a median of −9.61°, 6° to 63° with a median of 29.78°, 5° to 43° with a median of 25.63°, 29° to 90° with a median of 55.41°, and −34 mm to 224 mm with a median of 37.95 mm, respectively. The mean NRS was 1.99 ± 0.729 (range: 1–5). All data are summarized in Table 1.

**TABLE 1:**
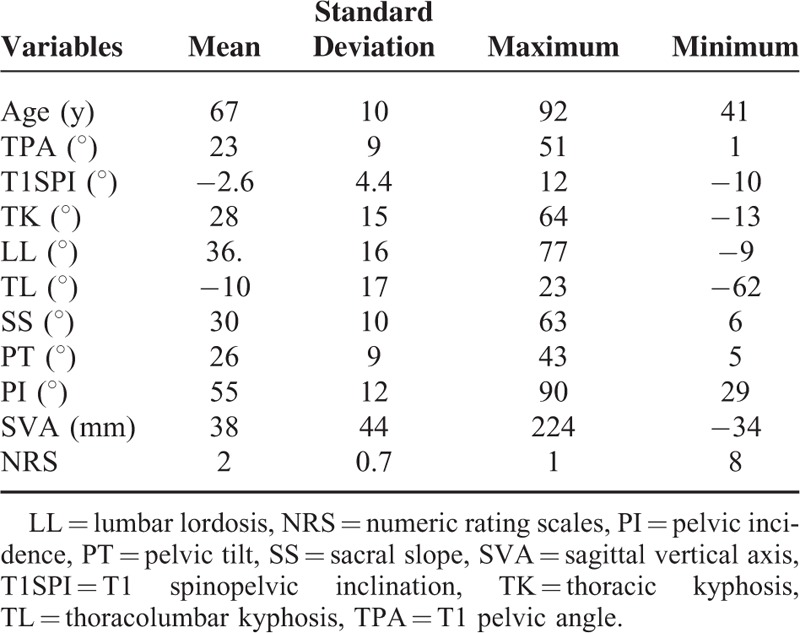
Demographic Characteristics, Sagittal Parameters, and Health-Related Quality of Life for Patients

The inter- and intraobserver reliabilities of the TPA measure had excellent agreement (ICC = 0.985 and 0.919, respectively). The correlation coefficient test showed that TPA was correlated with age (*r* = 0.318, *P* = 0.001), SVA (*r* = 0.624, *P* < 0.001), T1SPI (*r* = 0.424, *P* < 0.001), LL (*r* = −0.435, *P* < 0.001), PT (*r* = 0.884, *P* < 0.001), and PI (*r* = 0.482, *P* < 0.001), whereas no correlation was observed between TPA and TK, TL, and SS. In terms of health-related quality of life, TPA was also correlated with NRS (*r* = 0.557, *P* < 0.001). These associations were similar to those for age, LL, PT, PI, T1SPI, and SVA. The results of correlation coefficient test are summarized in Table 2.

**TABLE 2:**
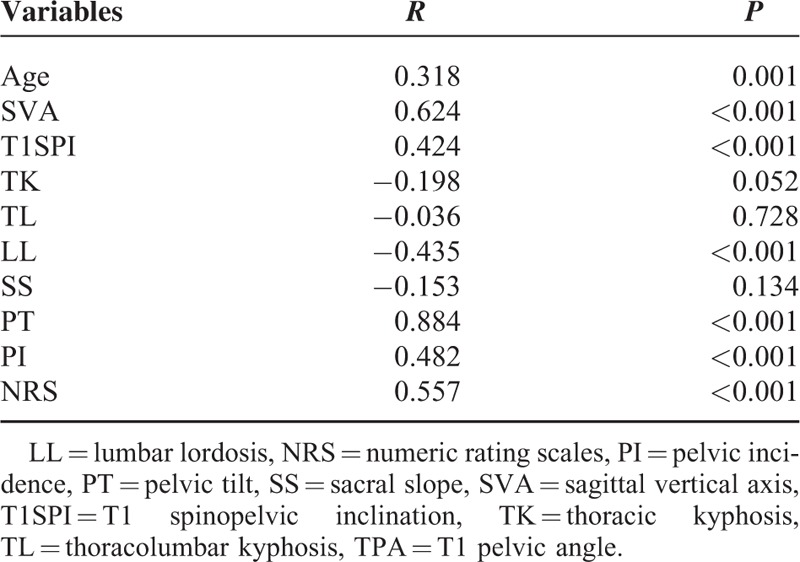
Correlations Between TPA and Other Sagittal Parameters and NRS

According to the threshold proposed by Ryan et al^5^, 38 patients with a mean SVA of 14.11 mm were included in Group A (TPA ≤ 20°), and 59 patients with a mean SVA of 53.31 mm were included in Group B (TPA > 20°). Of the 38 well-aligned patients, SVA was ≤5 cm in 33 (86.84%) patients and >5 cm in the other 5 (13.16%) patients. Of the 59 poorly aligned patients in Group B, SVA was >5 cm in 42 (71.19%) patients and ≤5 cm in the other 17 (28.81%) patients. There were significant differences in age (*P* < 0.001), LL (*P* < 0.001), SS (*P* = 0.013), PT (*P* < 0.001), PI (*P* = 0.001), T1SPI (*P* = 0.007), SVA (*P* < 0.001), and NRS (*P* < 0.001) between the 2 groups of patients. However, there was no significant difference in TK (*P* = 0.252) and TL (*P* = 0.376) between them (Table 3).

**TABLE 3:**
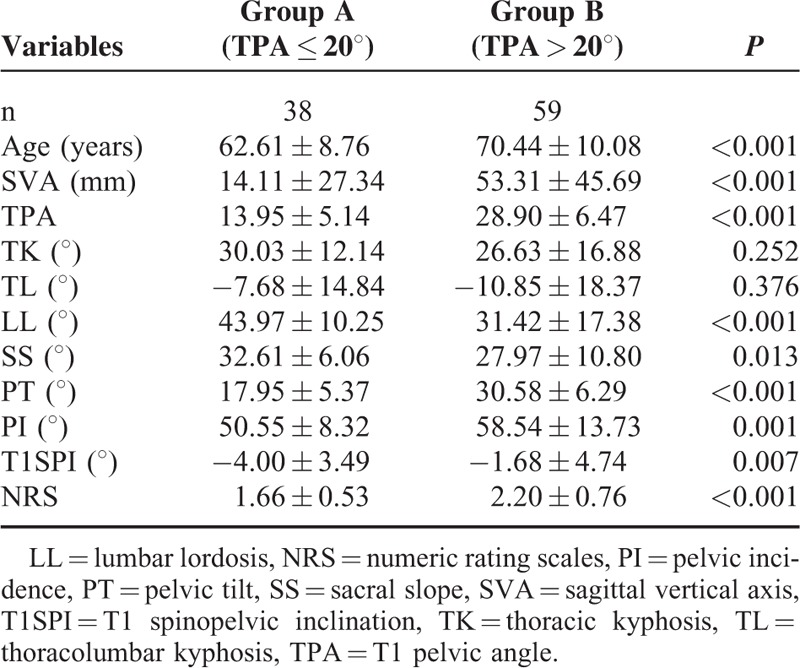
Comparison of Sagittal Parameters and Health-Related Quality of Life Between 2 Groups

## DISCUSSION

Low back pain afflicts ∼84% people in industrialized countries^9^. Recently, more attention has been paid to the relationship between low back pain and sagittal balance, because sagittal parameters have been proved to be significantly correlated with health-related quality of life measures.^2,3,15^ Sagittal balance is usually assessed by 2 categories of sagittal parameters: spinal parameters including SVA and T1SPI, and pelvic parameters including PT and PI. Of the spinal parameters, SVA is the most commonly used parameter to evaluate whether the spine of a patient is well-aligned or not. However, the disadvantages of SVA are being noticed due to the controversial results of some studies.^3,16^ Glassman et al^16^ suggested that the correlation coefficient between spinal parameters and ODI (Oswestry Disability Index) was weak (SVA and ODI, *r* = 0.281, *P* < 0.001), whereas Lafage et al^3^ argued that the spinal parameters were highly correlated with ODI (T1SPI and ODI, *r* = 0.52, *P* < 0.001), leading to the postulation that errors in measuring the sagittal parameters such as SVA on lateral radiographs might contribute to this difference, which required calibration of the radiograph and could vary in different studies.^6^ In addition, spinal and pelvic parameters represent spinal balance and pelvic retroversion, respectively, and none of these parameters could be used to evaluate the whole sagittal balance.

To deal with this difficult problem, Protopsaltis et al^6^ introduced TPA as a novel angular measure of global sagittal alignment, reporting that TPA was related to both PT and SVA. Unlike SVA, TPA did not vary with the extent of pelvic retroversion or patient support in standing and therefore could be used as a preoperative planning tool. However, all the relevant studies on TPA focused on measuring sagittal deformity in ADS patients and assessing radiological and clinical outcomes longitudinally. Whether TPA could be used to assess sagittal balance in patients with unspecific pain remains unknown. More importantly, spinal and pelvic parameters vary with different ethnicities^8^ and the results of studies in Caucasian populations might not be suitable to be used in Asian populations.

To the best of our knowledge, no study has been conducted to determine whether or not TPA could be used to represent and assess sagittal balance in Chinese patients with unspecific low back pain effectively. The aim of this retrospective study was to investigate the feasibility of using TPA to measure sagittal balance in Chinese patients with unspecific low back pain and explore its correlation with health measures and other sagittal parameters in these patients.

It was found in our study that the mean TPA in Chinese patients with unspecific low back pain was 23.04° with the standard deviation of 9.45° versus 17.3° in the study of Protopsaltis et al^6^ and 29.3° in the study of Qiao et al.^7^ Our finding is inconsistent with prior studies, probably because of measurement errors arising from different sample sizes, different ethnicities, and different surgeons. We observed a significant correlation between TPA and age (*r* = 0.318, *P* = 0.001), SVA (*r* = 0.624, *P* < 0.001), T1SPI (*r* = 0.424, *P* < 0.001), LL (*r* = −0.435, *P* < 0.001), PT (*r* = 0.884, *P* < 0.001), and PI (*r* = 0.482, *P* < 0.001), indicating that TPA was highly correlated with SVA and T1SPI that represent the global sagittal balance, and PT and PI that were compensatory mechanisms to affect the magnitude of SVA. Besides, no correlation was observed in TK, TL, and SS. According to Protopsaltis et al, TPA was correlated with SVA (*r* = 0.837), PI-LL (*r* = 0.889), and PT (*r* = 0.933), and the correlation coefficient between TPA and SVA and SS was 0.832 and 0.649, respectively, as reported by Qiao et al.^7^ Although our study and the other 2 studies all discovered a correlation between TPA and SVA, the amplitude of correlation was not all the same, probably due to different sample sizes, different ethnicities of patients and measurement errors in assessing the angle on the radiographs. With regard to the health-related quality of life measures, our results suggest that TPA was correlated with NRS (*r* = 0.557, *P* < 0.001), indicating that TPA, as a parameter in the sagittal plane, which did not bear the error inherent in measuring offsets in noncalibrated radiographs, could well represent the function status of patients, which is consistent with several other studies.^2,3,6^

According to the threshold proposed by Ryan et al,^5^ of the 38 well-aligned patients in Group A, SVA was ≤5 cm in 33 (86.84%) patients and >5 cm in the other 5 patients (13.16%), and of the 59 poorly aligned patients in Group B, SVA was >5 cm in 42 (71.19%) patients and ≤5 cm in the other 17 (28.81%) patients. In both groups, most patients whose TPA was ≤20° or >20° were well-aligned (86.84%) or poorly aligned (71.19%) respectively, indicating that TPA could represent sagittal imbalance in patients with unspecific low back pain. Besides, there were significant differences in age, LL, SS, PT, PI, T1SPI, SVA, and NRS between the 2 groups, suggesting that this threshold could be used to assess differences in sagittal parameters and health-related quality of life measures between well-aligned and poorly aligned patients. Furthermore, our study showed an excellent agreement in inter- and intraobserver reliability of the TPA measure (ICC = 0.985 and 0.919, respectively), which is similar to the result of Protopsaltis et al, who reported that there was an excellent agreement in TPA (inter- and intraobserver reliability: ICC = 0.980 and 0.902, respectively) and it compared favorably with the inter- and intraobserver reliability for SVA (0.995 and 0.917), PT (0.959 and 0.853), and PI (0.909 and 0.866).

It was found in our study that TPA was correlated with SVA, NRS, and other sagittal parameters. In addition, it could represent sagittal imbalance in patients with unspecific low back pain and be used as a parameter to evaluate the whole sagittal balance. Therefore, TPA could be a substitute for SVA in light of the following advantages. First, TPA is an angle and does not require a calibrated image, which might be a source of measurement error. In addition, TPA combines both the information of SVA and PT, which could represent the whole sagittal balance. Finally, TPA is consistent in the same patient despite varying degrees of pelvic retroversion and knee flexion, which is immune to the posture of the patient.

Although we have obtained some meaningful results in this study, there are some limitations that are worth notice. First, all the recruited patients came from the outpatient clinic of our hospital, which might result in selection bias. Second, only 1 of the health-related quality of life (NRS) was used to assess the pain of the patients without using other measurements such as ODI, which may possibly miss the relationship between TPA and the disability of the patient. Besides, selection and publication biases may not be avoided due to the retrospective nature of the study in a single center. Therefore, larger-scale and multicenter studies are required to verify our conclusion about the use of TPA in patients with unspecific low back pain.

## CONCLUSIONS

The present study has demonstrated a correlation between TPA and age, LL, PT, PI, T1SPI, SVA, and NRS, and excellent reliability of TPA as well. Given the significant differences in sagittal parameters between well-aligned and poorly aligned patients, TPA could be used to assess sagittal balance in outpatients with unspecific low back pain effectively.
